# `An observational report of intensive robotic and manual gait training in sub-acute stroke

**DOI:** 10.1186/1743-0003-9-13

**Published:** 2012-02-13

**Authors:** Lucas Conesa, Úrsula Costa, Eva Morales, Dylan J Edwards, Mar Cortes, Daniel León, Montserrat Bernabeu, Josep Medina

**Affiliations:** 1 Functional Rehabilitation Department, Neurorehabilitation Hospital Institut Guttmann, Badalona, Barcelona, Spain; 2 Brain Injury Unit, Neurorehabilitation Hospital Institut Guttmann, Badalona, Barcelona, Spain; 3 Non-invasive Brain Stimulation and Human Motor Control laboratory, Burke Medical Research Institute, White Plains, New York, USA; 4 Berenson-Allen Center for Non-Invasive Brain Stimulation, Harvard Medical School, Boston, MA, USA

**Keywords:** Gait training, stroke, body weight support, rehabilitation

## Abstract

**Background:**

The use of automated electromechanical devices for gait training in neurological patients is increasing, yet the functional outcomes of well-defined training programs using these devices and the characteristics of patients that would most benefit are seldom reported in the literature. In an observational study of functional outcomes, we aimed to provide a benchmark for expected change in gait function in early stroke patients, from an intensive inpatient rehabilitation program including both robotic and manual gait training.

**Methods:**

We followed 103 sub-acute stroke patients who met the clinical inclusion criteria for Body Weight Supported Robotic Gait Training (BWSRGT). Patients completed an intensive 8-week gait-training program comprising robotic gait training (weeks 0-4) followed by manual gait training (weeks 4-8). A change in clinical function was determined by the following assessments taken at 0, 4 and 8 weeks (baseline, mid-point and end-point respectively): Functional Ambulatory Categories (FAC), 10 m Walking Test (10 MWT), and Tinetti Gait and Balance Scales.

**Results:**

Over half of the patients made a clinically meaningful improvement on the Tinetti Gait Scale (> 3 points) and Tinetti Balance Scale (> 5 points), while over 80% of the patients increased at least 1 point on the FAC scale (0-5) and improved walking speed by more than 0.2 m/s. Patients responded positively in gait function regardless of variables gender, age, aetiology (hemorrhagic/ischemic), and affected hemisphere. The most robust and significant change was observed for patients in the FAC categories two and three. The therapy was well tolerated and no patients withdrew for factors related to the type or intensity of training.

**Conclusions:**

Eight-weeks of intensive rehabilitation including robotic and manual gait training was well tolerated by early stroke patients, and was associated with significant gains in function. Patients with mid-level gait dysfunction showed the most robust improvement following robotic training.

## Background

The recovery of independent walking is one of the major goals of rehabilitation after stroke, remaining as a leading cause of serious long-term disability[1]. More than 30% of patients who have had a stroke do not achieve a complete motor recovery after the rehabilitation process[2,3]. For this reason, new rehabilitation approaches are needed in order to improve quality of life in stroke patients.

There is no unique approach for rehabilitation of gait after stroke[4]. From physical therapy interventions (such as Bobath, Perfetti, Propioceptive Neuromuscular Facilitation - PNF) [5,6] to more technological approaches including the use of Functional Electric Stimulation (FES) [7] or Body Weight Support Robotic Gait Training (BWSRGT),[8-10] many therapeutic options have been used alone and combined to improve motor recovery in stroke. The beneficial effects of treadmill training have been extensively investigated in the last fifteen years in stroke patients,[11] including the greater effects of the body weight support training[12,13]. Many studies have reported that electromechanical devices have at least the same efficacy as manual gait training with less effort by the patient and physiotherapist[14-17]. In a recent study, electromechanical assisted gait training has been shown to improve the independent walking ability (FAC) but not the walking speed when compared sub-acute and chronic patients that received conventional gait training[8].

The clinical characteristics of patients who benefit most from BWSRGT are presently unclear. If clinical variables that predispose to a positive response to BWSRGT could be identified, it might be possible to individually tailor the rehabilitation program and include BWSRGT for those patients who would have the greatest benefit in the rehabilitation process[18-20].

The treatment dose (number of hours of therapy and frequency) has been reported as a valuable variable of outcome,[21] but the optimal dose is still uncertain. Robotic gait training using the BWSGT for 4 weeks (5 ×/week) has previously shown in a small number of early stroke patients to be well tolerated, and is reported to improve gait function[22]. We applied a daily intensive program with robotic gait training in our inpatient setting of sub-acute stroke patients, and then followed with a period of consolidation using manual gait training. We report our observations of functional gain in a large number of patients by using accepted clinical scales to measure gait speed, assistance in locomotion and balance. The main goal of this study is to assist with clinical decision making and to power randomized controlled clinical trials.

## Methods

### Subjects

103 subjects with sub-acute stroke (< 6 months post-stroke) were followed in a prospective observational study from March 2006 to March 2009. Thirty-four patients did not complete the 8-week program due to factors not related to the study such as hospital discharge, or medical complications (such as pneumonia or infections). Of the 69 patients who finished the training protocol (49 men, 20 women, mean age 48 ± 11 years), 36 suffered hemorrhagic stroke and 33 ischemic stroke. According to residual deficits, 34 patients presented right hemiparesis, 28 patients presented left hemiparesis and 7 presented tetraparesis. 85.5% of the patients were non-ambulatory (FAC ≤ 2, n = 59) and 14.5% were ambulatory patients (FAC ≥ 3, n = 10). Mean post-stroke interval was 72 ± 38 days (Table 1).

**Table 1:** Patient baseline characteristics.

Baseline characteristics of stroke patients (n = 69)		
**Age (yrs)**		48 ± 11

**Days since stroke**		72 ± 38

**Gender**	Female	20

	Male	49

**Affected side**	Right hemiparesis	34

	Left hemiparesis	28

	Tetraparesis	7

**Stroke lesion**	Ischemic	33

	Hemorrhagic	36

**FAC initial**		1.30 ± 1.23

	Non-ambulatory (≤ 2)	59

	Ambulatory (≥ 3)	10

**Tinnetti Balance initial**		6.14 ± 3.84

**Tinnetti Gait initial**		4.10 ± 3.10

**10 MWT initial**		0.17 ± 0.22

Baseline demographic and functional characteristics of the stroke patients enrolled in the 8-week intensive rehabilitation program.

Data collection was obtained from patients who were performing BWSRGT program at the Neurorehabilitation Hospital Institut Guttmann (Badalona, Spain) following the clinical protocols and according to the local Ethics Committee. All patients gave written informed consent prior to enrolment in the study.

Stroke patients were included in the study if they were within 180 days post-stroke, presented clinical hemiparesis or tetraparesis, aged 25-75, were able to voluntarily participate into the study, were not expected to be discharged in the next 8-weeks and had the ability to perform manual gait training with or without external devices (initial FAC 0-4).

They were excluded if they had severe cognitive and/or language deficits that precluded them from following instructions, had contraindication for physical exercise (unstable cardiac status, or other pre-morbid condition that discard them from rehabilitation), had severe spasticity that interferes with robotic function or/and rigid join contractures/malformations on the lower limbs (> 10 degrees), or had inability to stand even in parallel bars.

### Training Intervention and Inpatient Rehabilitation

The robotic and manual gait training intervention was part of a comprehensive intensive rehabilitation program consisting of 5 hours per day/5 days per week. The rehabilitation program included occupational therapy, physical therapy, gait training, fitness, sports therapy, hydrotherapy and other activities oriented to achieve the maximum degree of functional independence (including urban tours or cooking training).

**Figure 1:**
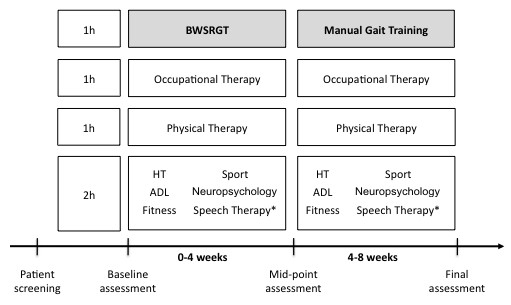
**Schematic of the intensive rehabilitation program**. The rehabilitation period comprised 8 weeks (5 hrs/day, 5 days/week); the first 4 weeks with Body-Weight-Supported-Robotic-Gait Training (BWSRGT) and the last 4 weeks Manual Gait Training. (*) Depending on patient individual needs and clinical goals.

Patients performed two contiguous blocks of 4-weeks of intensive rehabilitation program. The duration of each block period was based on previous literature[23,24]. During the first period, patients performed robotic body weight supported gait training (BWSRGT, described below), while in the second 4-week period the BWSRGT was substituted for manual gait training (MGT). In each case the gait training was performed daily as part of the 5-hour rehabilitation activities (Figure 1).

**Figure 2:**
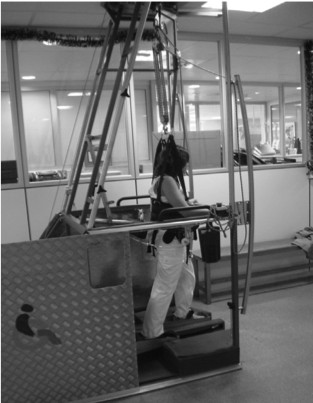
**A sub-acute stroke patient during the Robotic Gait training session**. During the Body-Weight-Supported-Robotic-Gait Training (BWSRGT) the body weight is slightly unloaded via the use of a harness, while the fixed foot placement on the device ensures a set pattern that mimics human gait with alternate stance and swing phase.

The BWSRGT was performed with a Gait Trainer^® ^(Reha-Stim, Berlin), where patient is held by a harness with each foot attached to a footplate that moves to mechanically simulate the stance and swing phase of gait controlling the ankle angle at push off and heel strike (Figure 2). The duration of the exercise ranged from 20-40 min depending on the tolerance of the patients (when patient reported excessive fatigue the training would stop). The percentage of weight unloaded varied between patients and corresponded to the weight that allowed the patient to stand with complete knee extension. Velocity ranged between 0.28 meter per second (m/s) and 0.42 m/s (to prevent overexertion of the patient). Step length was adjusted to the available range-of-motion for each patient. An elastic strap was placed on the paretic knee to help extension during the complete stance phase if needed (as a passive support).

The manual gait training (MGT) consisted of gait training over ground, with technical aids and the support of a physiotherapist as needed. Conventional technical aids for stroke patients are considered unilateral, ie knee-ankle-foot orthosis (KAFO), ankle foot orthosis (AFO), dynamic ankle foot orthosis (DAFO) and functional electrical stimulation (FES). To provide more stability, crutches, walkers (in tetraparesic patients), or parallel bars were allowed. Gait training was done under the supervision of a physical therapist, who provided verbal instructions and physical assistance to facilitate the swing phase when needed.

Our work intends to provide benchmark clinical gains data and tolerability from this atypically intensive rehabilitation program of consecutive blocks of robotic and manual gait training.

### Functional Outcome Assessment

The functional outcome assessment battery comprised: Functional Ambulatory Category (FAC),[25] Tinetti Balance and Gait Scale[26] and 10 Meter Walking Test[27]. Each outcome was assessed at baseline, mid-point, and at the end of the study (0, 4, 8 weeks) by an experienced physiotherapist.

The *FAC *was performed to assess gait ability and autonomy[28]. This ordinary scale includes 6 levels ranging from 0 to 5. FAC = 0 means no ability to walk or needed 2 assistants to help them walk, FAC 1 = able to walk with the constant attention of one assistant, FAC 2 = able to walk with someone for balance support, FAC 3 = able to walk with one assistant beside them to give them confidence, FAC 4 = independent walking but need some help with stairs or uneven ground, FAC 5 = independent for gait function in any given place.

The *10 MWT *quantifies walking speed, step length and cadence[29]. Patients were permitted to use technical aids during the test. This test was performed three times per evaluation and the mean speed was calculated. Patient gait was videotaped for extra documentation.

The *Tinetti Gait and Balance Scale *examines gait pattern and balance level[30]. The gait subscale ranges from 0 to 12, zero indicates and inability to walk or unable to perform any of the events of the gait pattern correctly, and 12 indicates a correct gait pattern. The balance subscale ranges from 0 to 16, zero indicates very poor balance and 16 good control of the equilibrium.

Our centre considers a clinically meaningful change in function to correspond with approximately 2 points in the FAC, 0.20 m/s in the walking speed, 3 points in Tinetti Gait Scale and 5 points in the Tinetti balance test.

### Data analysis

In this observational prospective study we used categorical variables described by frequency and percentage, and continuous variables described by mean and standard deviation (mean ± SD). Median and inter-quartile range (IQR) was used to explore asymmetry when necessary. The non-parametric Friedman test was used to assess differences between the 3 assessment measures, while the non-parametric Kruskall-Wallis test was used to assess outcome based on initial functional level. The alpha level was set at p < 0.05.

## Results and Discussion

**Figure 3:**
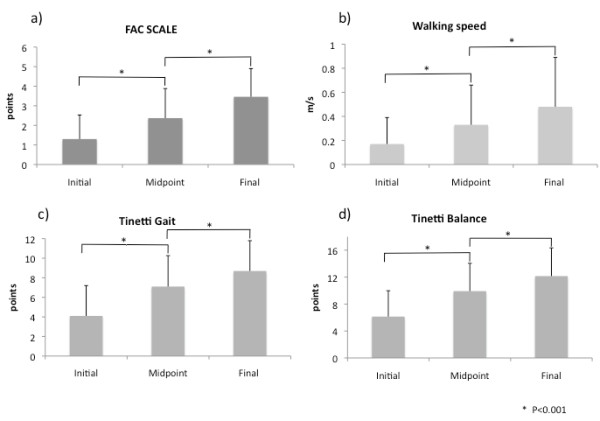
**Functional outcome results**. Improvement in functional outcome across the robotic and manual training period (mean ± SD). For each outcome: (a) FAC (b) walking speed (c) Tinetti Gait and (d) Tinetti Balance, there was a significant increase following the robotic training, and further consolidation from the follow-up manual gait training.

We report results from 69 patients who completed the intensive training program, showing significant improvements for each outcome (Figure 3).

### Functional Ambulatory Category

Across the 8-week intensive rehabilitation period FAC score improved by 45% across the period of robotic training (baseline 1.30 points ± 1.23, mid-point 2.37 ± 1.51, p < 0.001), and improved by a further 31% following the manual training (final assessment 3.14 ± 1.52, p < 0.001). 88% of patients improved by one point or more on the FAC scale across the 8-week intervention period.

### Walking speed

Walking speed improved by 46% across the period of robotic training (baseline 0.17 m/s ± 0.22, mid-point 0.33 ± 0.33, p < 0.001), and improved by a further 22% following the manual training (final 0.48 ± 0.41, p < 0.001). Across the full training period 83% of patients improved more than 0.20 m/s.

### Tinetti Gait Scale

Tinetti Gait Scale improved by 45% across the period of robotic training (baseline 4.10 ± 3.10, mid-point 7.10 ± 3.16, p < 0.001), and improved by a further 18% following the manual training (final 8.69 ± 3.10, p < 0.001). During the 8-week rehabilitation period 56% of patients improved more than 3 points on Tinetti Gait Scale.

### Tinetti Balance Scale

The Tinetti Balance Scale score improved by 38% across the period of robotic training (baseline 6.14 ± 3.84, mid-point 9.92 ± 4.13, p < 0.001), and it improved by a further 18% following the manual training (final 12.15 ± 4.19, p < 0.001). Across the 8-week rehabilitation period 65% of patients improved more than 5 points.

### Patient demographic and initial clinical status

The functional changes according to age, gender, initial FAC, initial Gait and Balance Tinetti and initial walking speed, were analyzed during the 4-week BWSRGT period, the 4-week manual gait training period and at the 8-week total intensive rehabilitation period. Patients with an initial FAC of 2 or 3 showed significantly greater changes in walking speed than patients with other initial FAC across the 4-week robotic training protocol (Table 2). These changes were maintained over 4 weeks of manual gait training. The change in each outcome was not significantly influenced by variables; gender, age, aetiology (hemorrhagic/ischemic), affected hemisphere, initial walking speed, initial Balance Tinetti and initial Gait Tinetti.

**Table 2:** Change in outcome measures stratified by initial Functional Ambulatory Category (FAC).

		Change 0-4 weeks BWSRGT			Change 4-8 weeks MGT			Change 0-8 weeks BWSRGT+MGT		
**Initial FAC**	**n**	**Gait Tinetti**	**Balance Tinnetti**	**Gait Speed**	**Gait Tinetti**	**Balance Tinnetti**	**Gait Speed**	**Gait Tinetti**	**Balance Tinnetti**	**Gait Speed**

0	19	2.00 ± 1.8	1.69 ± 1.7	0.11 ± 0.16	2.77 ± 2.7	3.08 ± 3.4	0.08 ± 0.19	4.76 ± 3.60	4.77 ± 3.70	0.22 ± 0.36
1	27	1.28 ± 1.4	2.72 ± 1.8	0.10 ± 0.16	3.83 ± 2.5	4.11 ± 2.5	0.16 ± 0.16	5.11 ± 2.76	6.83 ± 3.16	0.28 ± 0.27
2	13	2.29 ± 0.7	2.71 ± 1.4	0.09 ± 0.14*	3.29 ± 1.2	4.71 ± 0.7	0.29 ± 0.33	5.57 ± 1.13	7.43 ± 0.97	0.48 ± 0.52
3	5	1.50 ± 1.3	1.75 ± 2.8	0.04 ± 0.27*	1.00 ± 0.8	2.50 ± 1.7	0.23 ± 0.23	2.50 ± 1.29	4.25 ± 3.30	0.28 ± 0.18
4	5	1.00 ± 0.7	2.00 ± 2.3	0.12 ± 0.09	2.00 ± 1.4	3.60 ± 3.0	0.62 ± 0.14	3.00 ± 1.58	5.60 ± 3.36	0.18 ± 0.14

The table describes the change in the Tinetti Gait, Tinetti Balance, and Walking Speed during the Body Weight Support Robotic Gait Trainer (BWSRGT) training (0-4 weeks); during the Manual Gait Training (MGT) period (4-8 weeks); and the change over the 8-weeks total of intensive rehabilitation period. Subjects with initial FAC 2 and 3 showed significant improvement in walking speed across the robotic training period. (*) p < 0.05.

During the 8-week training period the non-ambulatory patients (FAC ≤ 2) went from 85% (n = 59) at baseline to 34% (n = 24) to the end-point; and the ambulatory patients (FAC ≥ 3) went from 14% (n = 10) at baseline to 65% (n = 45) at the endpoint (Table 3).

**Table 3:** Number of patients in each Functional Ambulatory Category (FAC) over the treatment period.

FAC	Baseline Week-0	Mid-Point Week-4	End-Point Week-8
**0**	19	8	3
**1**	27	15	10
**2**	13	15	11
**3**	5	10	11
**4**	5	17	18
**5**	0	4	16

**FAC**	**Baseline** **Week-0**	**Mid-Point** **Week-4**	**End-Point** **Week-8**

**≤ 2 **(non-ambulatory)	59	38	24
**≥ 3 **(ambulatory)	10	31	45

At commencement of the study, the majority of patients were in low FAC levels, with nil patients in Category 5. This trend changed across the weeks to ultimately have a more even spread across categories at week 8, including 16 patients in the highest category and only 3 remaining in the lowest category. This trend is highlighted with division of the FAC into non-ambulatory (FAC ≤ 2) and ambulatory (FAC ≥ 3), showing a movement of majority from non-ambulatory (85% of the patients, n = 59) at commencement, to ambulatory by completion of the training program (65.2%, n = 45).

## Discussion

The results of this observational study provide evidence that a comprehensive and intensive eight-week rehabilitation program including BWSRGT followed by Manual Gait Training in patients early after stroke, can led to an improvement in all functional outcomes, independently of patient demographic or initial functional status. However, the results indicate that patients with initial FAC level of 2 or 3 obtained the most benefit. The intensive rehabilitation program was well tolerated, and no patients withdrew for factors related to the gait training or the high training dose.

Other research groups have studied the improvement of walking ability of sub-acute stroke patients combining methods of gait training showing that an intensive locomotor training on an electromechanical gait trainer plus physiotherapy resulted in a significantly better gait ability and daily living competence compared with physiotherapy alone[22,31].

However, many studies have failed in showing significant differences in gain of functional scores when comparing robot-driven gait orthosis training with conventional physiotherapy[32]. In the study by Peraula et al,[33] chronic ambulatory patients regained the same walking ability when they received body weight supported training with or without FES compared with over-ground walking exercise training program.

The dose or intensity of the training seems to influence the improvement in the walking ability improvement since our study shows greater results than studies with only 20 or 30 min of daily therapy for 3 to 4 weeks[32,33]. Higher intensity of gait practice, in line with modern principles of motor learning, probably explains the superior results. The total amount of rehabilitation given to the patients is higher than reported in other studies[22,34] and our results should be considered in the context of high-intensity rehabilitation in sub-acute stroke.

In our study, after 8-weeks of intensive rehabilitation we found gait improvements in one or more of the outcome measures in 95.54% patients. This finding is higher than recovery reported by other authors[23,35] and may indicate that the higher dose in our rehabilitation program can lead to greater improvement of motor function in sub-acute stroke patients. To determine the magnitude of the improvement attributed to gait training, a comparison with an experimental control group without gait training would need to be done. This considered, the results of the present study should be used as a benchmark for expected change to aid clinical decision-making and to power controlled clinical research studies.

The selected functional outcome measures were sensitive to detect change across patients and may be suitable to use for future studies. Care should be taken interpreting functional scales that may include the use of assistive technology (as can be used in the 10 MWT). The underlying factors of patient performance leading to improved scores on each outcome measures is difficult to determine from the present study. For example, the improved score on the Tinetti balance test could be a cause of improved gait, since various balance functions are known to affect gait[36,37]. The more the patient sways, the worse is the balance and consequently the gait parameters[38]. According to Kollen et al[39] the recovery of independent gait is highly dependent on improvements in control of standing balance. These results are in line with our study, where the gait speed and functional ambulatory measures improve in parallel with balance measures.

A pertinent finding of our study is that patients with mid-range FAC at admission obtained the most benefit, which raises the possibility that FAC could be tested as a clinical predictor for recovery, although Masiero et al[20] did not find a correlation between FAC and motor recovery during conventional rehabilitation programs. Other studies have shown that initial level of paresis[40] or trunk control[41] could be used as clinical predictors of balance and gait for rehabilitation in sub-acute patients. Moreover, previous studies have reinforced the idea of the excellent reliability of the FAC, good predictive validity and responsiveness in sub-acute stroke patients and it has been proposed to predict community ambulation with high sensitivity and specificity[28]. We provided evidence that suggests that FAC could be useful as a predictor of outcome, but not initial walking speed, as reported by Barbeau[42].

One of the limitations of the present study is that the design of the study does not allow the comparison of the robotic gait training with the same amount - 60 min - of conventional gait therapy, combined both with an intensive rehabilitation program. The characteristics of the patients that would benefit the most of this type of combined gait therapies (robotic + conventional) remain unclear. In our study, patients ranged from the early phase of recovery to 3 months after the injury, when the largest gains are observable,[43-45] however some studies have found improvements in gait function in late phases of recovery[46]. The optimum dose of therapy is another open question for future studies. Even if daily therapy seems to be a decisive factor in the training program success early after stroke, [14,47] there is disparity of results about what is the ideal frequency of training in chronic phases[48,49].

## Conclusions

This study shows that gait training using the BWSRGT is associated with improved walking function in individuals with sub-acute stroke, and that it is feasible and safe to combine with a comprehensive and intensive functional rehabilitation program over 8-weeks. Further studies need to address if the improved walking parameters after a combined and intensive gait rehabilitation program are maintained over time. Moreover, the optimal dose of training characteristics (frequency and duration), as well as the precise gait parameters associated with training responsiveness, need further research.

## List of abbreviations

KAFO: knee-ankle-foot orthosis; AFO: ankle foot orthosis; FES: functional electrical stimulation; DAFO: dynamic ankle foot orthosis; FAC: Functional Ambulatory Categories, BWSRGT: Body weight supported robotic gait training.

## Competing interests

The authors have no competing interest in the present study. This work was supported by Spanish National Health Research Grant PI082004 (Beca FIS) and NIH grant 5R21HD060999-02 for DJ Edwards.

## Authors' contributions

LC, UC and EM contributed equally with data acquisition, analysis and interpretation as well as study design and manuscript development. DE and MC contributed with data interpretation, intellectual content and critical review of the manuscript. DL contributed with the revision of the last version of the manuscript. MB and JM contributed with the original study design, and manuscript development. All authors read and approved the final manuscript.
